# Woolsorters’ Disease—Structure of the Spermatozoa—Diphtheria Spread by a Cat—Eucalyptus Globulus—Depraved Taste in Animals—Bromide of Ethyl—Hens—Sheep’s Foot—Feeding Horses—Earthworms and Carbuncle, Etc.

**Published:** 1880-10

**Authors:** 


### PROGRESS OF VETERINARY SCIENCE.

WOOLSORTERS' DISEASE IN BRADFORD.—Some interesting facts have recently been brought to light with regard to the woolsorter's disease at Bradford, which are strongly confirmatory of the previous evidence that this disease is identical with anthrax or splenic fever of the lower animals.

Some weeks ago, during the time when several deaths occurred in the neighborhood of Bradford, one or two cases occurred at Harden hills, one at least being fatal. A week or two later a cow, which was grazing in a field adjoining the factory, died rather suddenly, and it was at that time suggested that it was a case of splenic fever, but the result of the post-mortem was doubtful. Since that time two sheep, grazing in the same field, have also died rather suddenly, and last week another cow was taken ill and died in four hours

A post-mortem examination, made by Dr. Bell, of Bradford, Dr. Roberts, of Keryhleg, and Mr. Collins, V. S, showed that the animal had died of splenic fever. Portions of the viscera were forwarded to Dr. Greenfield at the Brown Institution, and he confirmed the diagnosis.

It appears that the soap-water used for washing wool in the factory is conducted directly into the fields, and there distributed by means of trenches cut in the soil.

The cows drink the water readily. There seems little doubt that the disease is really derived from this source, and this is not only very important as evidence of the infectious character of the wool, but is conclusive proof that the washing is not conducted at a sufficiently high temperature to act as a disinfectant.

We learn that two other cows have since died in the same field with similar symptoms and post-mortem appearances. One or two more cases of the disease have occurred among the woolsorters. In one case of malignant pustule, the local serum proved to be infectious on inoculation, but the man recovered.

There seems every reason to believe that more stringent legislation will be the only means of checking the disease, the recent outcry having been apparently ineffectual in enforcing preventive measures.—*London Lancet.*

---

THE STRUCTURE OF THE SPERMATOZOA.—In the current number of the Quarterly Journal of Microscopical Science is a short paper by Dr. Heneage Gibbes, in which he states that he has found the spiral filament, discovered by him in the spermatozoa of several species of animals, as the rat, mouse, axolott, pigeon, fowl, snail, and leech. In the examination of different specimens of human spermatozoa, he has noticed a variation in the length of the tails, and in one specimen he found a number of heads with no corresponding tails He throws out the suggestion that these variations may have some important bearing It is quite possible that tailless spermatozoa may not be able to fertilize the ovum, while the greater the length of the tail, the greater their locomotion and fertilizing power may be.

Dr. O. S. Jensen, of Bergen, has found the spiral filament in the semen of horses; and Prof Fleming, of Kiel, has also confirmed Dr. Gibbes' observations, both as to the existence of this filament with its mesentery, and the different reaction to the staining fluids of the head and middle part of the spermatozoa.—*London Lancet.*

---

DIPHTHERIA SPREAD BY A CAT.—The following account is given as authentic: Three deaths from diphtheria have recently occurred in the family of Baldwin Gordon, who lives at the beach opposite Patchogue, L. I., under very remarkable circumstances. Some time ago a cat, which had been owned by a family, several members of which were suffering from diphtheria, was taken to the Gordon house. While playing with this cat, a little child of Mr. Gordon was bitten in one of its fingers. The wound caused intense pain, and was soon followed by a soreness and ulceration of the throat, which a physician pronounced to be diphtheria. Others of the family were taken with the disease, and two of the children died.

Mrs. Gordon, who was recovering from sickness, was seized with the disease and died. It is reported that still another member of the family has died.

Physicians believe that the cat was suffering from diphtheria when it bit the child. —*Medical Record*, September 25th, 1880.

---

EUCALYPTUS GLOBULUS —The St. Louis Clinical Record, for September, 1880, has an excellent article on the use of eucalyptus globulus as an antiseptic and substitute for carbolic acid. In the form of spray, when dissolved in pure alcohol, it can be used in all the stages of Lister's antiseptic dressing.

Eucalyptus has, as yet, received but little attention or recognition at the hands of veterinarians. It is one of the best febrifuge remedies at our command. As a remedy in various bronchial diseases of a mild type, it is exceedingly valuable. As an antiperiodic in miasmatic diseases it has no rival, except cinchona, to which drug it is very analogous.

It is a good diuretic as well as diaphoretic, and contains enough of the resinous and stimulant properties to make it of great value in vesical catarrh.

Its astringent, tonic, and antiseptic properties render it useful in a great variety of diseased conditions, particularly in affections of the mucus membranes.

As a stimulant to foul and gangrenous ulcers it has no superior.

Applied to fresh wounds, it relieves pain and prevents suppuration, and makes an elegant and convenient dressing, agreeable in smell, and when used as an antiseptic it destroys all fœtid odors without substituting one more offensive.

At present there are four preparations of eucalyptus used in medicine: the extract, fluid extract, the tincture, and the oil (eucalyptol).

Some of the fluid extracts are made wholly from the leaves, and are deficient in the resin, and devoid of the antiseptic property.

A good fluid extract should contain all the bases, neutral and active principles of the plant. All the fluid extracts are readily miscible with water, more freely so, how ever, when the water is rendered slightly alkaline. They can be used for spray or simple dressing, and can be used in any form desired. The oil is soluble in equal parts of alcohol, and is the most elegant form for use in surgical cases and as an antiseptic. The tincture and extract answer just as well for internal administration.

E. B.

---

THE subject of a depraved taste in animals is an interesting one, which has not been studied as much perhaps as it might. In human beings it would seem to depend on ill health of either body or mind, but in animals it would seem as if it might be present, and the animal enjoy good health. One remarkable instance in an herbivorous animal we can vouch for. It occurred in a sheep that had been shipped on board of one of the P. and O. steamers, to help to supply the kitchen on board; but, while fattening, it developed an inordinate taste for tobacco, which it would eat in any quantity that was given to it. It did not much care for cigars, and altogether objected to burnt ends; but it would greedily devour the half-chewed quid of a sailor, or a handful of roll tobacco. While chewing there was apparently no undue flow of saliva, and its taste was so peculiar that most of the passengers on board amused themselves by feeding it, to see for themselves if it were really so As a consequence, though in fair condition, the cook was afraid to kill the sheep, believing that the mutton would have a flavor of tobacco. Another very remarkable case has just been communicated to us by Mr. Francis Goodlake; this time a flesh-eating animal in the shape of a kitten, about five months old, who shows a passionate fondness for salads. It eats no end of sliced cucumber dressed with vinegar, even when hot with Cayenne pepper. After a little fencing it has eaten a piece of boiled beef with mustard. Its mother was at least once seen to eat a slice of cucumber which had salt, pepper, and vinegar on it. The kitten is in apparently good health, and its extraordinary taste is not easily accounted for. Even supposing it once got a feed of salmon mayonaise, why should it now select to prefer the dressing of the fish?—*English paper.*

---

BROMIDE OF ETHYL is used as substitute for chloroform with good results.

---

HENS often have a habit of biting and pulling their feathers, and greadily eating them until their bodies are bare. This practice, it is believed, is occasioned by a want of salt, as when salted food is given them they make no attempt to continue the habit. Salt pork, chopped fine and fed twice a week, has been adopted with success, while others put a teaspoonful of salt with two quarts of meal or shorts moistened, well mixed, and feed it about twice every week. Fowls, like human beings, to be healthy, must have a certain allowance of salt.—*Indiana Farmer*, Sept. 18, 1880.

---

THE SHEEP'S FOOT. CARE AND NEGLECT OF IT.—Foot-root is a most destructive disease of sheep. There is an incipient and easily preventable and curable form of this disease, and there is a malignant and contagious foot-rot, which infects and poisons the soil, and spreads sometimes with fearful effect, among large flocks, destroying the sheep by hundreds and thousands The malignant form grows out of the other, and it is questionable if it could not be preventend from spreading among the sheep, even from infected ground, if their feet were only in good condition. But the sheep's foot is seldom in good condition naturally, because the shepherd rarely thinks it necessary to examine it until something wrong is evident, from the lameness caused by it. Then precaution comes too late. The manner of growth of the sheep's foot is peculiar, and upon this depends its proclivity to damage and disease. The walls of the hoof grow from above downwards, meeting the growth of the sole at the junction; the outer layer of the former being produced indefinitely, and, if not worn away by contact with the ground, pass the sole and spread beyond it, turning under, and forming a loose covering, beneath which moisture, filth, sand, stones, and other foreign matter find a lodgment. These foreign matters soften the horn of the sole, or otherwise injure it, so that disorganization or destruction occurs, and carries the injury to the interior of the foot. Stones or gravel that may be inclosed under the excess of horn, press upon the softened sole and irritate the sensitive tissues under it, and although as yet no actual damage may have occurred, yet the sheep is unable to walk upon its feet, and moves about on its knees. When this is seen, no time should be lost in examining the flock, and remedying the mischief, while this can be easily done. The feet will probably appear with the walls of the foot having outgrown the sole, and not only turning under at the sides but turning up at the toes, thus preventing the natural us- of the feet. This is to be remedied by the use of toe-nippers, made especially for trimming the feet, and also by the use of the knife. The walls of the feet are trime med at the sides with a knife, and all superfluous horn is removed. The toes are clipped with the nippers, a pair of common pincers may be used, if the edges of the claws are filed and ground sharp. Neglect of these precautions has ruined many flocks, while the pastures have become so poisoned with the diseased and infectious matter that no healthy sheep could be kept upon them until after an interval sufficient to rid them of the contagion. The result of neglect may be described as fol lows: The horn of the sole being softened and decomposed, as previously mentioned, and the sensitive inner portions of the foot being injured, inflammatory and suppurative action is caused within the foot; escape of the products of inflammation being impossible through the sole at first, intense suffering results, and a generally disturbed condition of the animal ensues. This is the first stage of malignant foot rot. In course of time the sole is decomposed, and fetid pus escapes, by which herbage and soil are infected. The disease spreads through the whole foot, and appears at the coronet. Fungoid, or mushroom-like excrescences appear on the sole and at the coronet, and, if neglected at this stage, the whole foot may be lost and the sheep ruined. In this condition, radical treatment is needed. The sound animals should be removed at once to new and clean pastures, or into a clean yard. The diseased sheep are to be treated by means of caustic dressings of the feet; hydrochloric (muriatic) acid, diluted with three times its bulk of water; a solution of one drachm of chloride of zinc in a pint of water, or carbolic acid should be used to destroy the diseased growth, and persevered in until sound parts are reached, when the usual stimulant dressing may be substituted. The sheep should be kept on a clean floor, covered or well dusted with air-slacked lime, or in a dry, clean soft pasture which should be ploughed as soon as its use by the sheep is no longer necessary.—*Mirror and Farmer*, Sept. 18, 1880.

---

AT a recent meeting of the New York Pathological Society, Dr. Delavan presented the heart of a setter dog, in the muscular substance of which there were nine large-sized buckshot. As the dog had not hunted for a number of years, it was fair to presume that the shot had been carried for a long period.—*Medical Record.*

---

SALICYLIC ACID, given at intervals of an hour for four hours and followed by castor oil in full doses, succeeded in expelling tania which had resisted all other remedies. One case reported had resisted all other medication for nine years.—*Louisville Medical Herald*

---

A SURGEON in the German army calls the attention of all who have to do with horses to the danger of using the pocket handkerchief to wipe away any foam from the mouth or nose of a horse which may have been thrown upon their clothes. Some months ago, the writer states, an officer came to him suffering from an obstinate cold and cough. The usual remedies were prescribed, but in vain; a visit to the baths at Reichenhall also did the patient no good. Returning to duty, the officer became worse; fever, attended with great pain in and swelling of the head, set in, and ultimately, after much suffering, he died with every symptom of glanders. Inquiries were set on foot, and it was found that, some time before he was taken ill, he had ordered a horse, which he believed was suffering from glanders, to be shot. Neither the groom nor any of the other soldiers who had been near the horse have been attacked by glanders, and, consequently, it is suspected that the officer who died may have conveyed the disease into his system by, perhaps, using his handkerchief to wipe some of the foam from the mouth or nose of the horse from his uniform.

---

A New Insecticide.—A German gardener has found by experience that black or green flies, caterpillars, etc , are at once destroyed by syringing the plants affected by them with water, in which the stems of the tomato plant have been well boiled. The liquor is applied when cold, and not only kills the insects, but leaves an odor which prevents others from coming —*American Naturalist.*

---

It is estimated that sheep to the value of $30,000 were killed by dogs in Kentucky in 1879. [Castrate all the dogs running at large. See Article IX., page 87, in No. 2 of the Archives.—Ed.]

---

Feeding Horses.—Much economy remains to be made in the feeding of horses. It is stated that, in this sense, forty per cent. of the rations of the French cavalry could be saved and the national budget lightened by several millions. M. Bouley, the celebrated veterinarian, attributes the salutary effects of oats on horses to an aromatic odor contained in the husk. Good oats, we know, ought to be dry, heavy, shining, slipping readily through the fingers, and exempt from all odor. An animal derives most benefit from his feed when he eats tranquilly and alone. He is inclined to bolt the grain when in company. The oats when bruised are more relished than when pounded. The new practice of regulating the feed by weight instead of by measure, is now becoming general. In Prussia, each regiment purchases its own fodder, and care is taken to reject oats that have sprouted from lying in sheaves on the soil till it has received rain. This detestable practice is the consequence of the difficulty of threshing oats with the flail when not slightly germinated. Horses fed exclusively on hay lack energy and vigor. Green soiling is not suited for those employed in hard work. Lucerne and clover are better green fodder than vetches and maize. The latter are more suitable for cattle. Every animal requires a certain volume of food to maintain the natural distension of the stomach; that is to say, concentrated food alone could not sustain life. In Prussia rye straw is given to horses along with oats. They do not eat the straw willingly; so that, when the horses are pronounced unfit for service and are bought by farmers, they are constantly attacked by colics in receiving the new rations, because the stomach and intestines have been contracted by the barrack rations, and time is necessary to enable them to return to their normal volume. Hay is of different qualities, and the feed of oats ought to be proportionate to the quality of hay The latter, if from marshy land, is not equal in point of nutrition to good oaten straw. Hay from irrigated districts looks well, but it is not very nutritive The best comes from a calcareous soil of mixed grasses, mingled with aromatic plants. Rye straw is only good for litter; wheaten straw is relished by horses in the night, and eaten at all times Carrots are more refreshing than nourishing for horses; turnips are next to worthless, but Jerusalem artichokes are good. In Bavaria, post-horses are fed on potatoes in place of oats. If the former be mixed with bran, so much the better. Bran fattens rather than imparts vigor, and that from rye is preferred in Germany to that from wheat, and sells at a higher price even. For horses having but little time to feed, bread is excellent, but should never be given fresh. In some garrison towns, livery keepers contract for the refuse bread of the soldiers. Linseed cake dissolved in water is good for nursing mares; rape cake, when cheap, is given to horses. Respecting beech-mast cake, that cattle and sheep eat with such avidity, horses will not approach it; hence the remark, it is for them a "poison." The strangest of all diets is saw-dust, yet for the barge horses along the Moselle and the Saare it is daily mixed with the feed of oats. It is slightly nutritive, but its efficacy lies in its maintaining the necessary expansion, or volume, of the stomach.—*French Cor. Am. Farmer.*

---

EARTHWORMS AND CARBUNCLE. (*Jour. de Méd. et de Chir. pratiques*, August, 1880.)—M. Pasteur, the great champion of the germ theory of disease, has studied the carbuncular disease (*charbon*) and malignant pustule most thoroughly, and has recently supplied a missing link in its history. It was found that the ground covering the bodies of animals dead of carbuncle was dangerous to sheep and cattle grazing in that neighborhood. Examination of the ground demonstrated the presence of bacteria and germ corpuscles, for many months, which were capable of infecting animals inoculated with them with carbuncle. In the month of August, 1878, a sheep that had died of carbuncle was buried in a farm garden at Béance. Ten months, then fourteen months afterwards, earth taken from over the burial place contained germ corpuscles, which gave carbuncle to animals experimentally. This, although the earth had been taken from the surface, and, in the interval, the ground had not been disturbed. A similar experiment gave like results with earth taken from the surface under which a cow, dead of carbuncle, had been buried two meters (6½ feet). The difficulty of explaining the ascent of these microscopic germs for great distances and against the flow of rainwater, etc., was apparently insurmountable. It was found that these parasites were contained in the little cylinders of earth voided by the earth-worms upon the surface of the gronnd, to which they ascend in the dews of morning or after rains. Among other germs, of septicæmia, of putrefaction, etc , those of this terrible disease were identified. The dust resulting from the disintegration of these cylinders is spread about upon the herbage, and thus it happens that grazing animals take it into their bodies and along with it the germs by means of which they become infected. In soils in which earth-worms do not thrive, the burial of infected bodies is harmless to living animals. It is needless to add that M. Pasteur sustained his assertions by incontrovertible proofs before the Academy of Medicine.—*St. Louis Clinical Record.*

---

AT a show of birds lately held in Berlin, several canaries were exhibited that attracted much attention on account of the peculiar colors of their plumage. Some were green, others red and light brown, and others of a soft gray tint, while all differed more or less from the light yellow of the common bird. These variations of color were produced by the daily use of Cayenne pepper in the food of the birds. The pepper is given in small quantities at first, and the birds appear to like it, but the immediate effects are anything but pleasing to the beholder. The feathers soon begin to fall, giving the bird very much the appearance of moulting; in a short time, however, new feathers make their appearance, and it is then, as they attain full growth, that they exhibit the curious tints observed.

